# His Bundle Pacing and Left Bundle Branch Pacing in Patients with Heart Failure

**DOI:** 10.3390/biomedicines12102356

**Published:** 2024-10-16

**Authors:** Patrycja Paluszkiewicz, Adrian Martuszewski, Jadwiga Radziejewska, Jacek Zawadzki, Jacek Smereka, Jacek Gajek

**Affiliations:** 1 Department of Emergency Medical Service, Wroclaw Medical University, ul. Parkowa 34, 51-616 Wrocław, Poland; 2 Division of Environmental Health and Occupational Medicine, Department of Population Health, Wroclaw Medical University, Mikulicza-Radeckiego 7, 50-368 Wrocław, Poland; 3 Klodzko County Hospital, 57-300 Klodzko, Poland; 4 Department of Anesthesia, Critical Care and Rescue Medicine, Collegium Medicum, University in Zielona Góra, 95-020 Zielona Góre, Poland; jacekzawadzkimed@gmail.com; 5 Medical Faculty, Wrocław University of Science and Technology, 50-368 Wrocław, Poland; jacek.gajek@pwr.edu.pl

**Keywords:** His bundle pacing, left bundle branch pacing, heart failure, QRS duration, stimulation threshold, cardiac resynchronization therapy, biventricular pacing

## Abstract

Background: His bundle pacing (HBP) and left bundle branch pacing (LBBP) are emerging therapies for patients with heart failure and conduction disorders, offering potential advantages over traditional pacing methods. These approaches aim to restore physiological conduction and improve cardiac function more effectively. Objective: This study aims to evaluate the efficacy and safety of HBP and LBBP in patients with heart failure and conduction disturbances, comparing these techniques to conventional pacing. Methods: A comprehensive review of recent studies and clinical trials was conducted, focusing on the performance of HBP and LBBP in improving cardiac function, reducing QRS duration, and enhancing overall patient outcomes. The analysis includes data on clinical efficacy, procedural safety, and long-term benefits associated with these pacing modalities. Results: Both HBP and LBBP have demonstrated significant improvements in cardiac function and clinical outcomes compared to conventional pacing. HBP effectively restores physiological conduction with improved synchronization and a reduction in QRS duration. LBBP has shown enhanced left ventricular activation, leading to better overall cardiac performance. Both techniques have been associated with a lower incidence of complications and a higher success rate in achieving optimal pacing thresholds. Conclusions: HBP and LBBP offer promising alternatives to traditional pacing for patients with heart failure and conduction disorders. These advanced pacing strategies provide superior clinical outcomes and improved cardiac function with reduced risk of complications. Further research and clinical trials are needed to fully establish the long-term benefits and safety profiles of these techniques in diverse patient populations.

## 1. Introduction

Heart failure (HF) is a syndrome of clinical symptoms most often presenting as shortness of breath due to congestion in the pulmonary circulation or edema caused by fluid accumulation. It may coexist with structural and functional abnormalities that can lead to reduced cardiac output and increased left ventricular filling pressure [1]. A significant group of HF patients consists of those with atrial fibrillation (AF), whose numbers continue to rise. Attempts to maintain or restore sinus rhythm in HF patients are usually ineffective. The worst prognosis is seen in patients with heart failure in NYHA class III and IV, where the percentage of patients with permanent AF reaches up to 60% [2,3]. In addition to rhythm disturbances, the development and progression of HF are also driven by specific electrophysiological disorders in the form of atrioventricular and intraventricular conduction abnormalities. These disorders may also be a consequence of HF, and their coexistence worsens prognosis and contributes to premature death [4].

The treatment of HF includes properly managed pharmacotherapy and the correction of electrophysiological disorders. The first important step in the pharmacotherapy of patients with heart failure is the implementation of loop and thiazide diuretics. Angiotensin-converting enzyme inhibitors (ACEI) have demonstrated effectiveness in reducing mortality, sudden cardiac death, and hospitalizations due to heart failure in patients with reduced left ventricular ejection fraction (HFrEF). As an alternative, angiotensin receptor blockers (ARBs) are recommended in cases of intolerance, and numerous randomized clinical trials (RCTs) confirm their efficacy in HFrEF. Candesartan significantly reduced the risk of cardiovascular death or HF hospitalization in patients with HFrEF, both with and without accompanying atrial fibrillation. Beta-blockers significantly reduce all-cause mortality, cardiovascular deaths, and hospitalizations in HF patients; however, they do not offer prognostic benefits in the presence of AF. Nonetheless, due to the lack of harm and the need for symptom or heart rate control, they should be used. After treatment with ACE inhibitors and beta-blockers, in patients with HFrEF and persistent symptoms (NYHA class II-IV), mineralocorticoid receptor antagonists (MRAs), such as spironolactone and eplerenone, are recommended. This pharmacotherapy is advised to mitigate adverse structural changes in HF. In atrial fibrillation, heart rate control is crucial, and for this purpose, beta-blockers—especially effective in high sympathetic tone—are used. Less commonly, digoxin is used, as well as non-dihydropyridine calcium channel blockers, which are not recommended in cases of significant HFrEF. The target heart rate is <110 beats per minute at rest, and this is more of a disease indicator than a therapeutic goal [3].

The criterion determining the choice of electrotherapy variant is the width of the QRS complex. Patients with a narrow QRS do not require resynchronization therapy and are eligible for the implantation of a cardioverter-defibrillator (ICD) when the left ventricular ejection fraction (LVEF) is 35% or lower [5]. In most patients, single-chamber systems are implanted, while dual-chamber systems are used for patients requiring atrial stimulation. If the probable percentage of right ventricular (RV) stimulation (>40%) is high, patients are qualified for resynchronization therapy with defibrillation function (CRT-D) [6]. ICD systems serve as a preventive measure against sudden cardiac death. Among patients with narrow QRS and persistent atrial fibrillation, a single-chamber ICD is typically implanted in cases of reduced LVEF, although this therapeutic approach does not contribute to heart rhythm regulation and requires the use of subtherapeutic doses of beta-blockers to avoid the harmful effects of high RV pacing rates and worsening left ventricular systolic function [7]. His bundle pacing and conversion to a dual-chamber ICD, which are permissible in the guidelines [8], offer significant therapeutic possibilities that provide an advantage over standard treatment and may, in selected cases, reverse the unfavorable remodeling of the left ventricle [9]. This procedure, previously considered difficult to perform, is gaining increasing popularity and seems to be the future of electrotherapy for patients with HF, narrow QRS complexes, and persistent AF [10].

In the group of patients with narrow QRS complexes and HFpEF or HFmEF with atrioventricular conduction disturbances, including those with a long first-degree atrioventricular block, resynchronization pacing can be considered. The guidelines note that the implantation of a cardiac pacemaker in these patients carries the risk of worsening left ventricular contractile function due to 100% ventricular pacing. By implanting a three-chamber device instead of a two-chamber one, we gain the ability to maintain the native morphology of the QRS complexes when using HBP. Standard resynchronization with left ventricular pacing from the epicardial vein is especially used in these patients when there is significantly impaired left ventricular function, but compared to HBP, where we preserve the native morphology of the QRS complex, it is less optimal and may even be detrimental [11].

Another group of patients consists of those with wide QRS complexes, for whom a resynchronization pacemaker or an ICD with resynchronization capability is used, depending on the baseline LVEF. In some patients with left bundle branch block (LBBB), particularly in the group with the best outcomes from electrotherapy, the implantation of an electrode for HBP can be considered as a method to bypass the LBBB due to its known ability to circumvent the site of damage in the cardiac conduction system and break the block. If it is not possible to achieve the resolution of intraventricular conduction disturbances and the results of left ventricular/epicardial pacing are suboptimal, the simultaneous pacing of the epicardial vein (a standard resynchronization element) and the His bundle region can be employed, which allows for better outcomes in the form of narrower QRS complexes through the simultaneous activation of the opposing walls of the left ventricle. This approach can easily be applied in patients with AF, where the port for the atrial electrode is used for HBP. This method of resynchronization was described in a study with the acronym HOT-CRT [12].

The prolonged presence of elements of the pacing system in the cardiovascular system, along with the implantation procedure itself, carries the risk of complications, including adverse effects of chronic pacing at the apex of the right ventricle, which can lead to the development of pacemaker-induced cardiomyopathy (PICM), changes in the histological structure of the cardiac muscle, disturbances in blood flow, and degenerative changes, increasing the risk of AF and mortality, as well as inducing or exacerbating HF [13,14].

Solutions to the problems associated with current methods seem to lie in techniques that ensure synchronized ventricular contraction and reduce the risk of complications related to pacing. The most physiological activation of the heart chambers is achieved through the propagation of impulses in the cardiac conduction system. For this reason, the future of electrophysiology lies in methods that stimulate the physiological conduction system [15].

The origins of cardiac electrotherapy can be traced back to the 1960s, when Paul Zoll successfully introduced external cardiac pacing, which was crucial for managing bradycardia and cardiac arrest [16]. Following this, the development of transvenous pacing in the late 1960s allowed for more effective and reliable pacing methods, significantly improving patient outcomes. By the 1980s, the advent of implantable ICDs revolutionized the treatment of life-threatening arrhythmias, marking a significant advancement in cardiac care and expanding the scope of electrotherapy. As early as the 1990s, studies were conducted on the effectiveness of modifications to the transvenous electrode configuration, demonstrating that the addition of a single-element subcutaneous array electrode did not increase the defibrillation threshold and did not cause complications related to the subcutaneous electrode during an observation period of 15.8 ± 2 months, showing the usefulness and safety of a single-finger array lead in lowering the defibrillation threshold [17]. At that time, the effectiveness of left ventricular (LV) pacing in patients with severe HF and LBBB was also being studied. The method using permanent epicardial electrodes showed high operative mortality. The transvenous method became preferable, but the catheterization of the coronary sinus and one of its branches presented a technical challenge [18].

**Figure 1:**
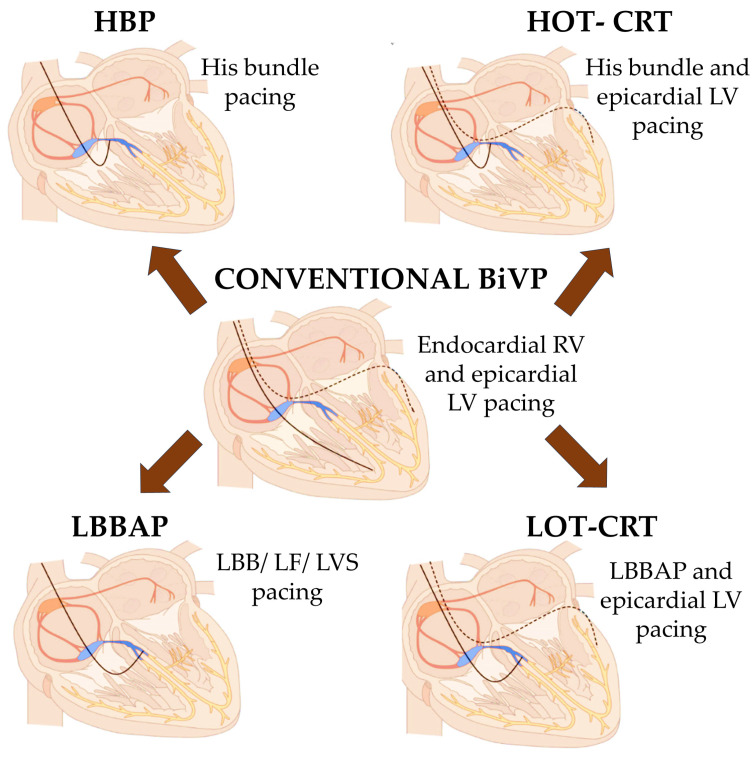
The methods of cardiac pacing are presented above.

In recent years, cardiac electrotherapy has focused on more physiological pacing techniques. Modern electrotherapy methods include HBP, left bundle branch pacing (LBBP), pacing in the area of the left bundle branch (LBBAP), and modifications of previously used pacing systems, such as His-Optimized Cardiac Resynchronization Therapy (HOT-CRT) and LBBAP-Optimized CRT (LOT-CRT) [19]. Figure 1 schematically presents the methods of cardiac electrotherapy, developed based on [20].

In the American guidelines from 2018 regarding cardiac pacing, HBP is mentioned alongside resynchronization therapy as a rational alternative to RVP in patients with AV block and an EF of 36–50%, where 40% RVP is expected, and as the sole form of pacing in other patients with a block at the atrioventricular junction [21]. HBP also represents an opportunity for patients with HFpEF, contributing to improved heart function [22]. Any method utilizing physiological pacing, compared to standard methods, leads to significant improvements in EF, a reduction in left ventricular size, and an improvement in functional class according to the NYHA classification [23]. The future of cardiac electrotherapy lies in further advancements in pacemaker and defibrillator technology, with an emphasis on minimally invasive techniques, wireless pacing, and remotely managed devices. Therefore, continued research into modern methods and the technology of physiological conduction system pacing, taking into account benefits and side effects, is essential for the development of cardiac electrotherapy.

## 2. Materials and Methods

We used the PubMed database to review the literature. We searched the database twice. The first search was for the literature on His bundle pacing in patients with heart failure. For this, we used the advanced search: “(his pacing) AND (heart failure)”. We applied filters: Full Text, Clinical Study, Clinical Trial, Clinical Trial Protocol, Clinical Trial, Phase I, Clinical Trial, Phase II, Clinical Trial, Phase III, Clinical Trial, Phase IV, Controlled Clinical Trial, Meta-Analysis, Multicenter Study, Observational Study, Randomized Controlled Trial, in the last 10 years. The search results showed 84 publications.

The second search was for the literature on left bundle branch pacing in patients with heart failure. For this, we used the advanced search: “(left bundle branch) AND (heart failure)”. These were the filters used: Full Text, Clinical Study, Clinical Trial, Clinical Trial Protocol, Clinical Trial, Phase I, Clinical Trial, Phase II, Clinical Trial, Phase III, Clinical Trial, Phase IV, Controlled Clinical Trial, Meta-Analysis, Multicenter Study, Observational Study, Randomized Controlled Trial, in the last 10 years. The search results showed 137 publications.

The third search was for the literature on modifications using bundle branch or left bundle branch pacing in patients with heart failure: HOT-CRT and LOT-CRT. For this, we used the advanced search: “((HOT-CRT) OR (LOT-CRT)) AND (heart failure)”. These were the filters used: Full Text, Clinical Study, Clinical Trial, Clinical Trial Protocol, Clinical Trial, Phase I, Clinical Trial, Phase II, Clinical Trial, Phase III, Clinical Trial, Phase IV, Controlled Clinical Trial, Meta-Analysis, Multicenter Study, Observational Study, Randomized Controlled Trial, in the last 10 years. The search results showed 4 publications.

In this systematic review, scientific articles published as of 30 July 2024, and covering the last 10 years, were used. In the course of compiling the collected literature, we excluded 67 papers for reasons of duplicate and 56 works were rejected due to incompatibility with the subject of this article. The creation of this publication adhered to PRISMA guidelines and registration information.

**Figure 2:**
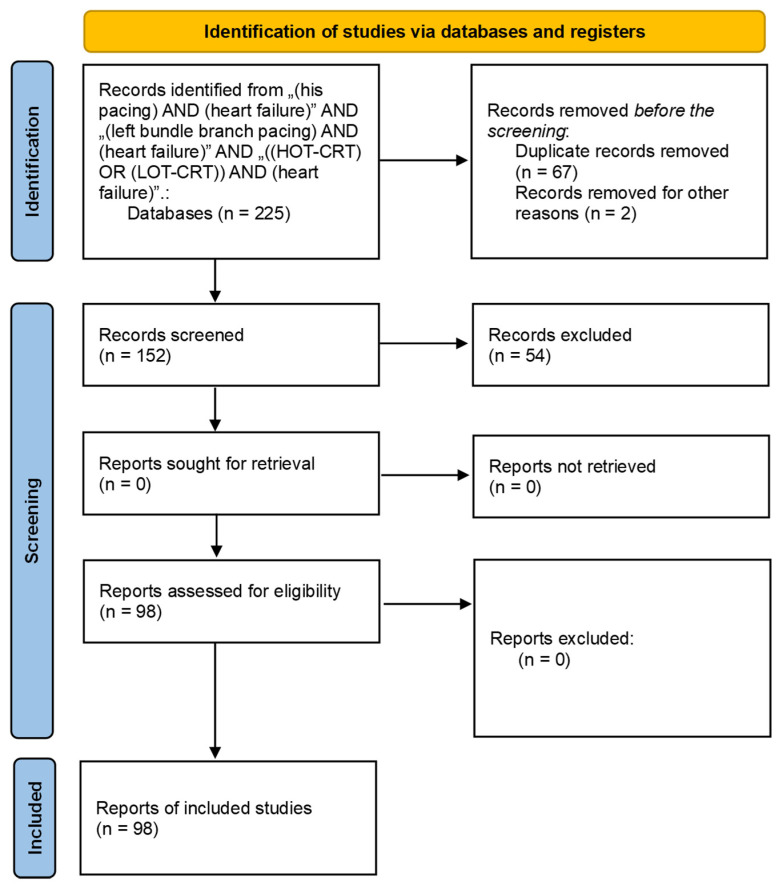
The flowchart presents the procedure for creating materials and the methodology of this scientific work.

In Figure 2, we present a flowchart of the procedure for the creation of materials and the methodology of this scientific work.

Table 1 presents 98 studies analyzed in this systematic review.

## 3. Candidates for Cardiac Resynchronization Therapy with Heart Failure and Atrial Fibrillation Who Undergo Atrioventricular Node Ablation

**Figure 3:**
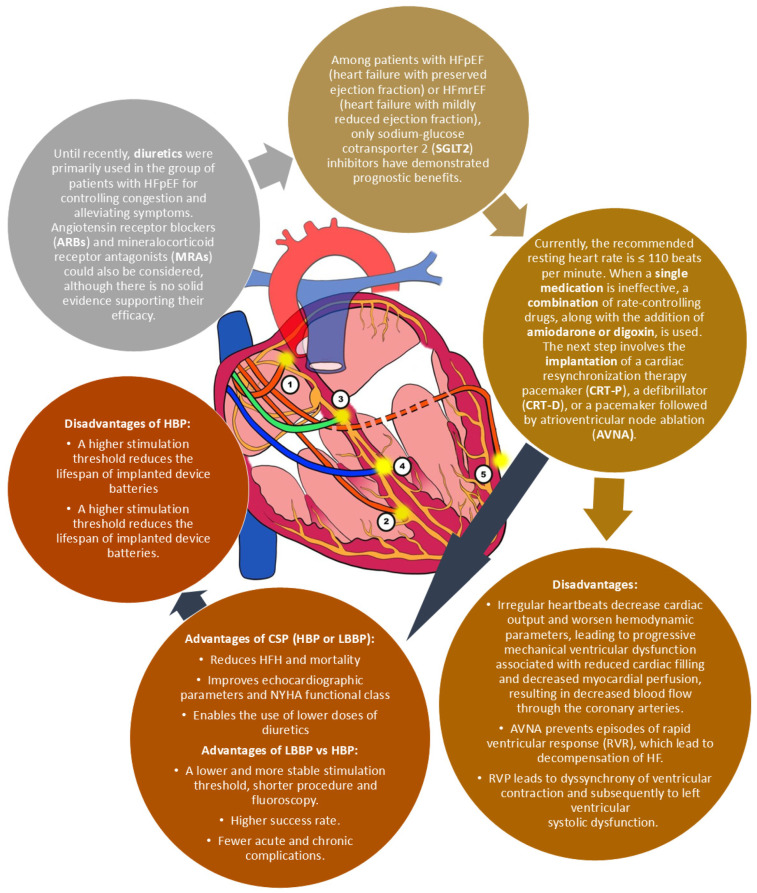
Comparison of first-line treatment AF with AVNA and CSP in patients with HFpEF, HFmrEF, and AF. Illustration [120] shows (1) atrial pacing; (2) right ventricular pacing (RVP); (3) His bundle pacing (HBP); (4) left bundle branch area pacing (LBBAP); (5) biventricular pacing (BVP) with an epimyocardial left ventricular lead via the coronary sinus (CS).

AF coexists in 6% of patients with NYHA class I and 15–35% of patients with NYHA class II-IV. It is a factor that worsens the prognosis in patients diagnosed with HF. Currently, there are no effective treatment methods for patients with permanent AF and heart failure with preserved ejection fraction (HFpEF) or heart failure with mildly reduced ejection fraction (HFmrEF). The PACE-FIB study aims to investigate the efficacy of rhythm control using LBBAP with atrioventricular node ablation (AVNA) in the aforementioned group of patients. This is a prospective, multicenter, open-label, randomized clinical trial planned from 2022 to 2027 with a 3-year follow-up period. The study group will consist of 334 patients with HFpEF/ HFmrEF and permanent AF who will receive LBBP followed by AVNA. The control group will consist of patients with pharmacological rhythm control [24]. Below is a comparison between conventional first-line treatment and innovative treatment (Figure 3). AVNA with conduction system pacing (CSP), especially LBBP, appears to be a promising treatment strategy in patients with HFpEF and HFmrEF. The limitation of new treatment methods is the availability of pacemakers and electrodes.

In another retrospective observational cohort study, the efficacy of LBBAP with AVNA was investigated as a treatment for AF resistant to pharmacological therapy in patients with advanced lung disease and AF. The study assessed structural changes in echocardiographic evaluations before and after the procedure, hospitalization rates, and the medications used. The findings showed similar echocardiographic parameters before and after pacemaker implantation, a significant reduction in rate control medications post-procedure, a notable decrease in heart failure hospitalizations (HFH) for LBBAP, and no significant complications. Thus, the study demonstrated the safety and efficacy of LBBAP with AVNA in treating AF in patients with advanced lung disease [25].

As many as 30 percent of patients with heart failure and left ventricular dyssynchrony do not benefit from cardiac resynchronization therapy (CRT). An alternative to this method is HBP. A study by Qian et al. [26] examined the efficacy of His bundle pacing in patients with HF using an assessment of QRS complex duration, effective pacing rate, pacing threshold, initial LV function, and mortality rate. The analysis included 11 studies with a total study group of 494 patients, and the inclusion criteria were patients with cardiomyopathy with AF undergoing AVNA and candidates for resynchronization therapy. It was demonstrated that permanent HBP shortened the duration of the QRS complex among those with indications for CRT from 165.4 ± 8.7 ms at baseline to 116.9 ± 15.8 ms after HBP (*p* < 0.0001). After bundle branch pacing, there was a significant increase in capture and bundle branch block correction thresholds (*p* < 0.05), a significant increase in left ventricular ejection fraction (LVEF) from 36.9 ± 3.3% at baseline to 48.1 ± 3.0% at follow-up (*p* < 0.0001, I2 = 88%), and left ventricular end-diastolic diameter (LVEDD) decreased from 58.2 ± 1.7 mm at baseline to 52.8 ± 1.7 mm at follow-up (*p* < 0.0001, I2 = 69%). There were also significant improvements in clinical parameters, such as a decrease in NYHA classification from 2.8 ± 0.4 at baseline to 1.6 ± 0.4 at follow-up (*p* < 0.0001, I2 = 92%) and a decrease in BNP levels from 609.3 ± 67.1 pg/mL at baseline to 216.6 ± 99.2 pg/mL after HBP (*p* < 0.001, I2 = 60%). HBP effects normal electrical and mechanical cardiac synchronization, but the long-term monitoring of the safety of the therapy and the pacing threshold is necessary. Randomized trials are needed to evaluate the efficacy of HPB in patients with HF.

A multicenter prospective randomized controlled trial has also been initiated among patients with HFpEF and recurrent AF, registered under the number ChiCTR1900027723. Due to the lack of an effective treatment method for patients with persistent AF and HFpEF, especially in the case of recurrent AF after ablation, Zhang et al. [27] decided to evaluate a new treatment strategy for patients with HFpEF and persistent AF who had undergone at least two catheter ablation attempts with recurrence of AF, followed by physiological conduction reconstruction after AVNA or pharmacological therapy to control the patient’s ventricular rate and maintain a regular heart rhythm. This strategy has been named His-Purkinje conduction system pacing (HPCSP) with AVNA. The primary endpoints will include differences in cardiovascular events and composite clinical endpoints (overall mortality) between the HPCSP group and the pharmacologically treated group. Secondary endpoints will include echocardiographic parameters, HFH, 6 min walk distance, and NT-proBNP levels. The main objective of the study is to provide evidence of clinical improvement and enhanced quality of life for patients as a result of HPCSP with AVNA.

A randomized controlled trial compared the treatment outcomes of HBP and biventricular pacing (BVP) in a group of patients after AVNA due to persistent AF and with a LVEF < 40% [28]. Each patient underwent AVNA and received both BVP and HBP. There was a significant improvement in LVEF after HBP compared to BVP. There was a significant improvement in LVEDD, B-type natriuretic peptide level, and NYHA class for both pacing methods, with no significant differences observed between BVP and HBP. However, long-term studies involving larger patient groups are needed. Among patients with persistent AF and high rates of ventricular pacing, LBBAP has an impact on the reverse remodeling of the left atrium [29].

In one of the prospective multicenter observational studies, the efficacy of BVP was compared to conduction system pacing in a group of 373 patients eligible for ablation and pacing (A&P). It was proven that LBBAP, compared to BVP and HBP, is associated with the shortest mean fluoroscopy and procedure time (*p* < 0.05), the lowest acute capture thresholds (*p* < 0.05), the lowest capture threshold after 12 months, and the longest estimated battery life (*p* < 0.05). Certainly, an advantage of LBBAP is its positive impact on device longevity. However, there were no significant differences in complication risk and HFH between the groups (*p* = 0.850) [30].

It is known that ablation and CSP in patients with symptomatic AF improve clinical outcomes; however, data on spontaneous sinus rhythm restoration (SSRR) are lacking. Based on a prospective observational study of patients with persistent AF and a drug-resistant high ventricular rate, a multivariate analysis was conducted, demonstrating the presence of independent predictors of SSRR: left atrial volume index < 49 mL/m² and duration of persistent AF <12 months. Among patients with both predictors, the rate of SSRR was 41.4% [31].

## 4. Patients with Heart Failure and Atrial Fibrillation Who Are Candidates for Cardiac Resynchronization Therapy

Wang et al. [29] also investigated the efficacy of LBBAP in patients with permanent AF requiring ventricular pacing and with LVEF > 35%, comparing it to standard right ventricular pacing (RVP). Over an average observation period of 13.9 ± 7.0 months, improvements were observed in LVEF (+0.7% vs. −2.2%, *p* = 0.007) and left atrial diameter (−1.63 mm vs. +1.23 mm, *p* = 0.011) for LBBAP compared to RVP. These results provide evidence of LBBAP’s role in the reverse remodeling of the left atrium and show clinical benefits for patients requiring a high percentage of ventricular pacing.

## 5. Patients with Atrioventricular Block and Heart Failure

One of the target patient groups that may benefit from LBBAP is those with atrioventricular block (AVB) and HF. From 1 January 2018, to 18 November 2021, a cohort observational study was conducted at West China Hospital involving 903 patients. This study was registered at ClinicalTrials.gov under the number NCT05722379. The study evaluated all-cause mortality, HFH, lead failure, periprocedure complications, cardiac death, and recurrent unexplained syncope. It demonstrated significantly lower risks of all-cause mortality, HFH, and recurrent unexplained syncope. Echocardiographic indices and left ventricular systolic dyssynchrony were better with LBBAP compared to RVP (*p* = 0.012) [32].

In a group of patients with HF and failed CRT, HBP holds great promise. In one study, HBP was used as the primary strategy in patients with AVB, bundle branch block (BBB), and a high rate of ventricular pacing as an alternative to BVP, and as a salvage strategy in cases where left ventricular (LV) lead implantation failed or there was a lack of response to BVP. The study demonstrated the utility of HBP both as an alternative to BVP and as a salvage strategy for unsuccessful BVP [33].

A similar goal was set by a single-center randomized controlled superiority trial registered under the number ChiCTR2000034335. This study included 210 patients with AVB and an indication for pacemaker implantation, conducted from January 2021 to December 2023. The evaluation in this study considered LVEF, NT-proBNP, LVEDD, distance covered in the 6 min walk test, quality of life (SF-36 scale), cardiovascular events, overall mortality, rehospitalization rate, and the incidence of major complications. The results of this study are currently being processed [34].

## 6. Patients with Heart Failure with Reduced Ejection Fraction and Left Bundle Branch Block

Between 2020 and 2022, a randomized controlled single-center study was conducted to evaluate the efficacy of LBBAP in patients with HFrEF and LBBB. The development of the LBBAP method aimed to address the shortcomings of BVP and HBP. The primary endpoint was the assessment of LVEF, while secondary endpoints included NT-proBNP, QRSd, LVEDD, left ventricular end-systolic diameter (LVESD), the 6 min walk test, quality of life, HFH, rates of major complications, length of hospital stay, and procedural costs. The control group consisted of patients treated with BVP. LBBAP was expected to provide benefits such as lower pacing thresholds, higher R-wave amplitude, and easier implantation [35]. It turns out that the presence of a scar at the implantation site is a determining factor for the success of performing LBBAP. High-sensitivity and high-specificity cardiac magnetic resonance imaging (CMRI) helps predict the success of therapy in patients with LV scarring using LBBAP [36]. LBBAP appears to be a safe and effective method even in patients over 80 years old. In addition to improving echocardiographic parameters, it has shown stable pacing thresholds during a 10-month observation period [37].

Mariani et al. [38] demonstrated that LBBP was associated with a significant reduction in HFH compared to BVP and HBP. However, the risk of lead revision was significantly higher for BVP and HBP compared to RVP. LBBP proved to be the best method in the study, maintaining lower pacing thresholds, higher R-wave amplitudes, and no increase in the risk of lead revision.

## 7. Patients with Bradycardia

In a group of patients with bradycardia, HBP and LBBP proved to be significantly better at maintaining physiological ventricular activation compared to RVP [39].

LBBAP appears to be a promising therapy for patients with bradyarrhythmia and conduction system disorders. While RVP worsens ventricular function, LBBAP preserves electrical and mechanical ventricular synchrony without differences in lead-related complications. LBBAP significantly reduces the risk of HFH, AF occurrence, and overall mortality compared to RVP, achieving similar pacing thresholds and higher R-wave amplitudes [40].

In another study, the LBBAP capture threshold was found to be significantly different, measuring 0.69 ± 0.39 V at 0.46 ± 0.15 ms, while in the case of HBP, it was 1.44 ± 1.03 V at 0.71 ± 0.33 ms (*p* < 0.001). An increase in the capture threshold was noted especially in HBP; however, there were no significant differences in adverse events between LBBAP and HBP. It has been assessed that CSP yields better results than standard CRT, and LBBAP can achieve better pacing parameters than HBP [41].

LBBAP is a promising method for treating bradyarrhythmias and HF. In the multicenter MELOS study, the efficacy and complications of different CSP sites were examined. The dominant type of LBBAP capture was left bundle fascicular capture (69.5%), followed by left ventricular septal capture (21.5%) and proximal left bundle branch capture (9%). Capture threshold and sensing were stable during follow-up. The study demonstrated the efficacy of LBBAP in the study group of patients in terms of shortening QRS duration and reducing LVEDD. The results also indicated that success rates and safety require further refinement [42].

## 8. Patients with Bradycardia and Conduction Disturbances

HBP and LBBP are promising treatments among patients with bradycardia and conduction disturbances. Currently, RVP is the most commonly used treatment in the aforementioned patient group. HPCSP overcomes the limitations of RVP.

One meta-analysis evaluated the results of treating 4160 patients with bradycardia and conduction disturbances with HBP, LBBP, and RVP. It was shown that RVP was associated with fewer lead-related complications and shorter procedure times than HPCSP, but resulted in decreased LVEF, prolonged QRS complex, and increased HFH compared with HPCSP [43].

Sun et al. [44] also compared HBP with RVP in a group of patients with bradycardia and conduction disorders. In a group of 2348 patients, they demonstrated a significant improvement in LVEF in HBP compared to RVP (mean difference [MD], 5.65; 95% CI, 4.38–6.92), significant shortening of the chronically paced QRS duration (MD, −39.29; 95% CI, −41.90 to −36.68), significant reduction in pacing threshold (MD, 0.8; 95% CI, 0.71–0.89), and a significantly lower risk of HFH (odds ratio [OR], 0.65; 95% CI, 0.44–0.96). No significant differences were found between the groups regarding left ventricular end-systolic volume (LVESV), left ventricular end-diastolic volume (LVEDV), and mortality. Therefore, HBP had an advantage over RVP among patients with bradycardia and heart conduction disorders.

One study aimed to determine whether BVP and/or HBP could prevent adverse remodeling and provide functional, structural, and clinical benefits compared to RVP in patients without severe left ventricular dysfunction (LVEF > 35%) and AVB. Patients with BVP or HBP showed significant reductions in LVESV and LVEDV (respectively, −2.77 mL, 95% CI −4.37 to −1.1 mL; *p* = 0.001; and −7.09 mL, 95% CI −11.27 to −2.91; *p* = 0.0009), as well as maintenance or improvement in LVEF compared to RVP (5.328%, 95% CI: 2.86–7.8%; *p* < 0.0001). The benefits of CSP were especially notable in patients with LVEF > 35% and ≤52%. Significant clinical improvement for BVP and HBP was primarily demonstrated in patients with chronic AF with rapid ventricular response, following AVNA and pacemaker implant [21].

In a study involving 703 patients with baseline bradycardia, LBBAP was compared to RVP. The endpoints included HFH, mortality, and conversion to BVP. In this patient group, LBBAP resulted in better clinical outcomes than RVP, including significantly narrower QRS duration, significant reductions in HFH and mortality, and a reduction in the primary endpoint among patients with an RV pacing percentage greater than 20% [45].

A retrospective study [46] in a group of 844 patients with indications for pacemaker implantation such as AVB, bradycardia with AF, and sinus node disease demonstrated the safety, efficacy, reliability, and better electrical performance of permanent HBP.

## 9. Patients with Atrioventricular Block and Differing Ejection Fraction

Another method using His bundle pacing is atrioventricular optimized His bundle pacing among patients with prolonged PR interval and heart failure with reduced ejection fraction who are not eligible for conventional biventricular pacing. In the HOPE-HF trial, the study group consisted of patients with heart failure, LVEF < 40%, PR interval > 200 ms, right bundle branch block (RBBB), and QRS < 140 ms. In total, 167 patients were implanted with an atrial pacing lead, a bundle branch pacing lead, and, depending on clinical indications, a cardioverter-defibrillator lead. Results showed no increase in LVEF with modified pacing compared with no pacing (+0.5%, 95% CI −0.7 to 1.6, *p* = 0.4) and no increase in peak oxygen uptake (+0.25 mL/kg/min, 95% [CI] −0.23 to +0.73, *p* = 0.3), while there was a significant improvement in patients’ quality of life as assessed by the Minnesota Living with Heart Failure Questionnaire (−3.7, 95% CI −7.1 to −0.3, *p* = 0.03). Bundle branch pacing optimized the time between atrial and ventricular contraction, and at the 6-month follow-up did not cause ventricular dyssynchrony or impaired left ventricular systolic function. However, long-term observations targeting endpoints are needed [47].

His bundle pacing is recommended to prevent the harmful effects of right ventricular pacing. Kronborg et al. [48], in a prospective randomized double-blind crossover study, evaluated LV function in a group of patients with atrioventricular block (AVB), narrow QRS, and preserved LVEF, who underwent HBP or para-HBP compared to right ventricular septal pacing (RVSP). They demonstrated significantly better LVEF and mechanical synchronization for HBP and para-HBP than for RVSP over a 12-month observation period.

His bundle pacing is also used in patients with advanced AVB and normal or mildly reduced ejection fraction (EF). In this group, HBP, biventricular pacing (BVP), and RVP were compared. The observation period ranged from 6 months to 5 years. HBP and BVP demonstrated significant increases in EF and reductions in QRS duration compared to RVP (*p* < 0.001), as well as significantly lower mortality and HFH rates. No significant differences were found between BVP and HBP. HBP and BVP proved to be superior methods for reducing the risk of HFH and mortality in patients with advanced AVB and normal or mildly reduced EF [49].

## 10. Patients with Heart Failure Left Bundle Branch Block Eligible for Cardiac Resynchronization Therapy

Right ventricular stimulation is a standard treatment method for individuals requiring ventricular pacing. However, the long-term stimulation of the right ventricular apex leads to significant complications, with the pathophysiology involving asymmetric hypertrophy and the dilation of the ventricles, abnormal alignment and fibrosis of myofibrils, asynchronous work of the heart chambers, structural changes, and a negative inotropic effect. Stimulating the elements of the physiological conduction system avoids the above changes; moreover, this approach can improve heart function. Sharma et al. [50] undertook the evaluation of the clinical efficacy, safety, and feasibility of permanent HBP compared to RVP in an unselected group of patients. The observation period was 2 years from the implantation of the pacemaker. It was demonstrated that pacing threshold (PTh) was significantly higher in the HBP group than in the RVP group and stable during the observation period (1.35 ± 0.9 V vs. 0.6 ± 0.5 V at 0.5 ms; *p* < 0.001). The HBP method was associated with significantly lower HFH compared to the RVP group (2% vs. 15%; *p* = 0.02) without a significant difference in mortality (13% in the HBP group vs. 18% in the RVP group; *p* = 0.45).

RVP causes heart failure and contributes to increased mortality. HBP as a method of physiological cardiac pacing reduces the number of deaths and hospitalizations due to HF becoming an alternative to the previously commonly used RVP. One study [51] compared the results of HBP with RVP among patients requiring pacemaker implantation. It found that HBP did not result in a decrease in LVEF, while a significant decrease occurred as a result of RVP. The HBP group compared to the RVP group showed a significantly lower incidence of pacing-induced cardiomyopathy (2% vs. 22%; *p* = 0.04), a significantly lower incidence of death or HFH (32% vs. 53%; HR: 1.9; *p* = 0.04), and a more frequent need for lead revision and generator replacement. Another study found an equivalent response between HBP and BVP. It is suspected that HBP may be more effective in patients with LBBB than previously thought [52]. Abdelrahman et al. [53] also evaluated the effectiveness of HBP in comparison to RVP, aiming to assess the primary outcome (death, HFH, or upgrade to BVP) and secondary endpoints (mortality and HFH). The primary endpoint was significantly better among patients with HBP compared to the RVP group (HR: 0.71; 95% CI: 0.534 to 0.944; *p* = 0.02), especially in patients with a ventricular pacing rate > 20%. A significantly lower rate of HFH (secondary endpoints) was found in HBP compared to RVP (12.4% vs. 17.6%; HR: 0.63; 95% CI: 0.430 to 0.931; *p* = 0.02). In summary, HBP was associated with a reduced risk of primary and secondary endpoints among patients requiring permanent pacing. Junior et al. [54] in their study also demonstrated the superior efficacy of HBP compared to BVP in terms of shortening the QRS complex, reducing LVESV, increasing LVEF, and improving NYHA class in patients with HF.

Due to complications and negative clinical outcomes, alternatives to biventricular pacing in CRT are currently being sought in patients with heart failure. Bundle branch pacing and left bundle branch pacing appear promising. They are part of the pacing system and provide physiological pacing and thus the physiological mechanical function of the heart. Gin et al. [55] conducted a meta-analysis based on 15 studies to compare the results of bundle branch/left branch pacing with biventricular pacing in patients with CRT convictions. Physiological pacing compared with BVP resulted in a reduction in QRS duration of 20.3 ms (95% CI: −26.1 to −14.5 ms; *p* < 0.05; I2 = 87.1%), an increase in LVEF of 5. 2% (95% CI: 3.5–6.9%; *p* < 0.05; I2 = 55.6), and a reduction in NYHA functional class of 0.40 (95% CI: −0.6 to −0.2; *p* < 0.05; I2 = 61.7). LBBAP compared with BVP was also shown to decrease the mean pacing threshold by −0.51 V (95% CI: −0.68 to −0.38 V), whereas HBP, compared with BVP, increased the pacing threshold (0.62 V; 95% CI −0.03 to 1.26 V). The results of the meta-analysis presented here are very promising for new methods of stimulation in the area of the pacing system. LBBAP can be achieved through LBBP or left ventricular septal pacing (LVSP), with LBBP generally yielding better results compared to LVSP [56]. The benefits of LBBAP were also described by Siranart et al. [57] and by Li et al. [121]. In one of the meta-analyses [58] examining the use of LBBP for CRT, a reduction in QRS duration from 172.7 ± 4.8 ms to 115.1 ± 7.6 ms was noted, along with improvements in LVEF and LVEDD over an average observation period of 8.1 months, with a complication rate of HFH at 1.3% and zero mortality. Another meta-analysis [59] demonstrated a significant reduction in HFH with the use of LBBP-CRT compared to BVP-CRT [7.9% vs. 14.5%; RR: 0.60 (95% CI: 0.39–0.93); *p* = 0.02], as well as a significant reduction in QRS duration, lower pacing threshold, improvement in LVEF, better response to therapy, and improvement in NYHA functional class. CSP brings electrocardiographic, echocardiographic, and clinical benefits [60,61,62].

In one study [63], the effects of implantation of an endocardial electrode on the LV side in the interventricular septum, known as LV septal (LVs) pacing, were compared with BVP and HBP for CRT. LVs pacing significantly shortened QRS duration and the standard deviation of activation times (SDAT) compared to BVP and LVs + RV pacing. However, the QRS duration, SDAT, and the first derivative of LV pressure (LVdP/dtmax) for LVs pacing were comparable to those for HBP.

An observational study [64] investigated the treatment outcomes of HBP and LBBAP compared to BVP among 477 patients with class I or II indications for CRT and with a LVEF ≤ 35%. Besides improved cardiac function, there was a significantly lower rate of heart failure hospitalizations and fewer deaths with CSP compared to BVP (28.3% vs. 38.4%; hazard ratio 1.52; 95% CI: 1.082–2.087; *p* = 0.013).

Similarly, Kim et al. [65] demonstrated that CSP significantly reduces overall mortality and HFH compared to biventricular pacing cardiac resynchronization therapy (BVP-CRT), but they emphasized the need for large randomized trials. Another study demonstrated comparable impacts on morbidity and mortality for LBBAP compared to BVP in CRT [66].

Wang et al. [67] also described the advantages of HPCSP involving HBP and LBBAP. They demonstrated that HPCSP compared to BVP in CRT improves electrocardiographic, echocardiographic, and clinical parameters, and reduces hospitalization rates and mortality in patients with an indication for CRT through physiological pacing of the stimulus–conduction system. Similar conclusions about the efficacy of HPCSP were drawn based on an observational study among patients with LVEF ≤ 35% and class I or II indications for CRT during an observation period of 27 ± 12 months [64].

Cheng et al. [68], in a meta-analysis examining the efficacy and safety of LBBP in patients with HF and LBBB, demonstrated that LBBP significantly reduced QRS duration and improved parameters such as LVEF, LVEDD, B-type natriuretic peptide levels, and NYHA functional class. Additionally, there was an increase in the pacing threshold by 0.06 V compared to RVP. However, randomized controlled trials are required to confirm these results.

The role of cardiac resynchronization therapy in HFmrEF is uncertain. The long-term outcomes of CRT in a group of patients with HFmrEF and LBBB were worse than in those without LBBB. LBBAP overcomes these limitations and is simpler and more affordable than biventricular pacing CRT. Researchers aimed to evaluate the effectiveness of LBBAP in HFmrEF as an alternative method to CRT. They demonstrated a significant improvement in LVEF from 39.8% to 50.5% (MD: 10.90%, 95% CI: 6.56–15.23, *p* < 0.01), and a significant reduction in QRS duration from 152.6 ms to 119.3 ms (MD: −34.51 ms, 95% CI: −60.00 to −9.02, *p* < 0.01). The study results provide evidence of improved systolic function in patients with LVEF between 35% and 50%, and support the use of LBBAP as an alternative strategy to standard CRT [69]. Baseline LBBB and LVEDD are independent predictors of echocardiographic response for LBBAP. Furthermore, the method provides stable and exceptionally low pacing thresholds [70].

Other parameters such as the standard deviation of time to peak velocity of 12 left ventricular segments (Ts-SD) for measuring mechanical synchrony and the maximum rate of left ventricular pressure rise (dP/dtmax) for assessing hemodynamics showed a greater reduction in Ts-SD (−14 ms [95% CI, −21 to −7 ms]; *p* = 0.001) and a significantly higher increase in dP/dtmax (6% [95% CI, 2–9%]; *p* = 0.002) for LBBP compared to BVP. LBBP has an advantage over BVP in terms of improving mechanical and electrical synchrony and cardiac hemodynamics in patients with heart failure and LBBB of ischemic etiology [71].

In a non-randomized observational study reported by Wu et al. [23], the results of treatment with CRT using LBBP to HBP and BVP were compared. The study group consisted of 137 patients with LVEF ≤ 40% and typical LBBB. In total, 135 of them received CRT from BVP, HBP, or LBBP. The mean duration of the paced QRS was, respectively, 135.4 ± 20.2 ms, 100.7 ± 15.3 ms and 110.8 ± 11.1 ms. HBP and LBBP showed similar absolute increases in LVEF after pacing (+23.9% vs. +24%, *p* = 0.977) and at the 1-year follow-up (74.4% vs. 70.0%, *p* = 0.881). In these cases, the LVEF was significantly higher compared to the BVP effect (∆LVEF +16.7% and 44.9% 1-year follow-up, *p* < 0.005). LBBP and HBP showed better improvement on the NYHA score compared to BVP. LBBP, compared to HBP, showed a higher R-wave amplitude (11.2 ± 41 5.1 mV vs. 3.8 ± 1.9 mV, *p* < 0.001) and a lower pacing threshold (0.49 ± 0.13 V/0.5 ms vs. 42 1.35 ± 0.73 V/0.5 ms, *p* < 0.001). LBBAP appears to be superior to HBP in terms of pacing parameters and implantation success rate as a first-line pacing strategy [72]. Despite the numerous benefits of LBBAP compared to BVP in CRT, the issue of complication risks for both methods cannot be overlooked. Among patients with NYHA class III and IV, BVP was associated with a lower rate of complication-free survival compared to LBBAP. Moreover, in multivariate analysis, BVP emerged as an independent predictive factor associated with a higher risk of complications (HR 3.234; *p* = 0.042). Up to 50% of all complications were related to the coronary sinus lead, most frequently observed with BVP-CRT [73].

When performing LBBP, an important and often overlooked aspect is the impact of the current of injury (COI) in the left bundle branch (LBB). In patients with positive COI (COI+), selective LBBP is more common than in patients with negative COI (COI-). Stimulation parameters are stable, and COI does not preclude safe LBBP stimulation. No perforations or lead displacements were observed [74].

Another publication compared the results of LBBP treatment with BVP treatment in CRT among patients with HFrEF. The LBBP procedure was assessed to take less time compared with BVP (MD 33.68 min, 95% CI: 17.80–49.55, I2 = 73%, *p* < 0.01) and required a shorter fluoroscopy time (MD: 9.68 min, 95% CI: 4.49–14.87, I2 = 95%, *p* < 0.01). As in other studies, a significantly greater reduction in QRS complex duration, a significantly greater improvement in LVEF, a greater reduction in LVEDD, a greater improvement in NYHA function class, and a lower risk of hospitalization for heart failure and mortality were reported for LBBP compared with BVP. However, the study included only nine observational studies and one randomized controlled trial. Further well-designed randomized controlled and observational studies are needed to thoroughly elucidate the long-term effects of LBBP treatment [75].

The rate of non-response to standard CRT remains high at 30–40%. Standard CRT through BVP employs non-physiological means, utilizing the fusion of an epicardial left ventricular wavefront with an endocardial wavefront from the right ventricular apex. The effectiveness of HBP in correcting the LBBB may stem from the pathophysiological basis of the LBBB pattern. LBBB is believed to be a disease located in the proximal part of the left conduction system. Proximal pacing provides an adequate stimulus that bypasses the site of the distal conduction block, thereby explaining the correction of the QRS complex. In the first randomized multicenter single-blind prospective controlled trial, the His-SYNC Pilot Trial [76], the outcomes of HBP used in place of a left ventricular lead in cardiac resynchronization therapy (His-CRT) were compared with biventricular pacing cardiac resynchronization therapy (BVP-CRT). The inclusion criteria for the study were standard indications for CRT. Therapy changes occurred in 48% of patients in the His-CRT group and 26% of patients in the BVP-CRT group. The most common reason for switching from His-CRT to BVP-CRT was the lack of correction of the QRS complex due to nonspecific intraventricular conduction delay, whereas His-CRT showed the significantly greater narrowing of the QRS complex compared to BVP-CRT (*p* < 0.001). No significant differences were found in HFH and mortality between the groups.

An alternative for patients who do not respond to CRT treatment is LBBP. In one study, the effectiveness of LBBP was evaluated among patients with CRT pacemakers or CRT defibrillators, who, after optimal pharmacological therapy and device optimization, showed a decrease in LVESV of less than 15% or an absolute increase in LVEF of less than 5%. The control group consisted of patients with BVP-CRT. In the LBBP group, LVEF significantly increased, while no significant improvement was observed in the BVP control group. Left ventricular end-diastolic volume and LBBB QRS morphology at baseline in a multiple logistic regression model were independent predictors of echocardiographic response after updating to LBBP. Additionally, the primary composite endpoint (death from any cause, heart-failure-related hospitalization events, or heart transplantation) was significantly lower in the LBBP group. The study provided evidence of the high effectiveness of LBBP in improving heart function and clinical outcomes [77]. Another multicenter retrospective study described by Ezzeddine et al. [78] assessed the outcome of CSP in 238 patients with HF compared with BVP on CRT using echocardiographic and clinical parameters. They defined response to CRT as an absolute increase in LVEF of >5% at 6 months after CRT. The response rate to CRT was significantly higher in the CSP group than in the BVP group (74% vs. 60%, respectively; *p* = 0.042). Kaplan–Meier analysis showed no significant differences in HFH time and overall survival using CSP compared with BVP (log-rank *p* = 0.78).

LBBAP may have a slight advantage over HBP in terms of stimulation threshold, fluoroscopy time, procedure time, and success rate [79].

It has been demonstrated that HBP has a lower complication rate, while LBBAP shows a higher success rate [80].

Similar conclusions were drawn in a study conducted by Tavolinejad et al. [81], where greater improvement in QRS duration, echocardiographic parameters, and clinical improvement were demonstrated in the case of conduction system pacing cardiac resynchronization therapy (CSP-CRT), while lower pacing thresholds were observed in the case of left bundle branch pacing cardiac resynchronization therapy (LBB-CRT) compared to standard biventricular pacing cardiac resynchronization therapy (BVP-CRT). Similarly, in a group of patients with heart failure, non-ischemic cardiomyopathy, and LBBB, LBBP-CRT demonstrated greater improvement in LVEF compared to BVP-CRT [82]. In heart failure patients with a reduced ejection fraction and wide QRS complexes, or where frequent ventricular pacing is expected, resynchronization therapy with biventricular pacing is the current treatment option. In an observational study, Vijayaraman et al. [83] showed that LBBAP is a good alternative to the aforementioned treatment. The study group was 981 patients with LVEF ≤ 35% after the first BVP and 797 patients with LVEF ≤ 35% after the first LBBAP. Time to death and hospitalization for heart failure and parameters assessed by echocardiography were taken into account. It was demonstrated that the duration of the QRS complex after LBBAP was significantly shorter than before pacing (128 ± 19 ms vs. 161 ± 28 ms; *p* < 0.001), and significantly shorter than after BVP (144 ± 23 ms; *p* < 0.001). Multivariate regression analysis showed a significant difference in LVEF improvement in favor of LBBAP compared with LVEF improvement after BVP (20.8% vs. 28%; HR: 1.495; 95% CI: 1.213–1.842; *p* < 0.001). The use of LBBAP was associated with lower mortality and less frequent hospitalization for heart failure compared to BVP.

Similarly, Al-Hennawi et al. [84] conducted a meta-analysis in a group of patients with HFrEF and indications for CRT comparing BVP, LBBP, and HBP. The shortest QRS complex duration was obtained with HBP [MD: −18.84 ms, 95% CI: −28.74 to −8.94; *p* = 0.0002]; the greatest improvement in LVEF was obtained with LBBP [MD: 5.74, 95% CI: 2.74 to 7.46; *p* < 0.0001], followed by the greatest improvement in LVEDD [MD: 5.74, 95% CI: 2.74 to 7.46; *p* < 0.0001] and NYHA class [MD: −0.58, 95% CI: −0.80 to −0.35; *p* < 0.00001].

In a group of patients with symptomatic HF and LBBB, BVP-CRT is a well-established treatment method aimed at correcting mechanical dyssynchrony due to delayed activation between the septal and lateral walls. The physiological correction of dyssynchrony is particularly desirable. Vinther et al. demonstrated that, in a group of patients with HF and LBBB, CRT using HBP yielded comparable clinical improvement to BVP, but at the cost of higher pacing thresholds [85].

Among the applications of HBP is the possible correction of LBBB in patients with HF. Huang et al. [86] conducted an observational study in the aforementioned group of patients, obtaining evidence of significant efficacy of HBP in terms of an increase in LVEF from 32.4 ± 8.9% to 55.9 ± 10.7% (*p* < 0.001), a decrease in LVESV from 137.9 ± 64.1 mL to 52.4 ± 32.6 mL (*p* < 0.001), and an improvement in NYHA class from a value of 2.73 ± 0.58 to 1.03 ± 0.18 (*p* < 0.001).

There have been many studies evaluating the negative effects of CRT with BVP among patients with HFrEF, LBBB, and LV systolic dysfunction. The benefits of BVP may be limited to patients with RBBB. One study [87] set out to test whether HBP is superior to BVP in CRT among RBBB patients with HF who are candidates for CRT. There was a significant shortening of the QRS complex and improvement in LVEF and NYHA class after HBP. The study highlighted the different physiology of HF with RBBB and HF with LBBB. With LBBB, the septum contracts earlier relative to the unactivated left ventricular wall manifested as asynchronous left ventricular contraction. With RBBB, there is asynchronous right ventricular contraction with normal left ventricular activation. The synchronization of delayed RV activation and normal LV activation can be achieved by RBBB recruitment with HBP or a combination of HBP (without RBBB recruitment) and RV pacing. No significant differences were found between the two synchronization methods, but HBP was shown to be beneficial for LV activation via the physiological pathway compared to BVP, resulting in prolonged LV activation time. In this study, both selective and non-selective His bundle pacing (NS-HBP) were used. In the absence of the correction of LBBB during NS-HBP, the duration of the QRS complex is longer than native. The fusion of NS-HBP and the right ventricular septum does not significantly narrow the delayed activation of the LV in LBBB. In the case of the RBBB, a fusion of NS-HBP and the right ventricular septum may partially or completely eliminate RV conduction delay without recruiting the right bundle branch (RBB); however, by pre-exciting the RV and allowing for the slow electrical activation of the RV, it may cause unfavorable hemodynamic effects. In the group of patients with NS-HBP and correction of RBBB with a small degree of RV fusion, hemodynamic effects may be minimal.

Chen et al. [88], in a prospective multicenter observational study on a group of 100 patients with HFrEF (EF ≤ 35%) and LBBB, compared the LBBP-CRT method with the optimized BVP with an adaptive algorithm (BVP-aCRT). LBBP-CRT is a method with a pacing electrode implanted in the LBB region and into the coronary sinus, while BVP-aCRT is a therapy using a pacing electrode implanted into the apex of the right ventricle and into the coronary sinus. QRS duration after pacing significantly differed in favor of LBBP-CRT compared to BVP-aCRT (126.54 ± 11.67 vs. 102.61 ± 9.66 ms, *p* < 0.001). Similarly, in the case of LVEF, significantly higher values after stimulation after the 6-month and 1-year of follow-ups were obtained by using LBBP-CRT compared to BVP-aCRT (respectively, 47.58 ± 12.02% vs. 41.24 ± 10.56%, *p* = 0.008 and 49.10 ± 10.43% vs. 43.62 ± 11.33%, *p* = 0.021). The LBBP-CRT method required a significantly lower pacing threshold compared with BVP-aCRT, both during pacemaker implantation and at the 1-year follow-up (*p* < 0.001).

Padeletti et al. [11] set out to investigate the outcomes of combined stimulation in CRT in a group of patients with HF and LBBB. Electrodes were placed in the right atrium, RV apex, coronary vein, and, additionally, in the His bundle. The following stimulation configurations were assessed: standard biventricular pacing (BVP; RV apex + LV), HBP only, LV only, and simultaneous HBP and LV pacing. Each of the methods was compared to AAI mode with multiple atrioventricular delays (AVD). In all groups, optimal AVD was close to the PR interval. Standard BVP significantly improved LVEF and reduced systolic dyssynchrony with individually optimized AVD. HBP + LV stimulation provided significant hemodynamic improvement, regardless of AVD settings. The study confirms the key role of intrinsic right ventricular conduction in optimal CRT.

Gould et al. [89] conducted a prospective multicenter randomized controlled trial to investigate whether triventricular pacing improves treatment outcomes compared to BVP-CRT among patients with LBBB and intermediate QRS prolongation (120–150 ms). Triventricular pacing involved the use of two LV electrodes. Despite atrioventricular pacing being performed without significant complications, a 6-month observation period did not show significant improvement in CRT response or clinical benefits following atrioventricular pacing.

The use of a His bundle pacing cardiac resynchronization therapy (HBP-CRT) lead instead of a lead placed in the coronary sinus results in improved clinical and echocardiographic parameters, as well as a 76% reduction in QRS duration [90].

Computer simulations were also used to evaluate CRT using HBP and LBBP. Unlike other studies, HBP and LBBP were compared to biventricular epicardial (BiV-epi) pacing and biventricular endocardial (BiV-endo) pacing. BiV-endo pacing was performed with a left ventricular (LV) lead at the lateral wall or with an LV lead at the LV septum. The study results showed that HBP was significantly better in terms of reduced LV activation times and inter-ventricular dyssynchrony compared to BiV-endo and BiV-epi (*p* < 0.05). LBBP, compared to BiV-endo and BiV-epi, also significantly reduced LV activation times but did not differ in terms of inter-ventricular dyssynchrony. However, optimizing AV delay reduced RV activation times and demonstrated a response comparable to HBP [91].

Another prospective multicenter observational non-randomized study compared LBBAP with BVP for CRT as an initial strategy for CRT. In a group of 371 patients, the study demonstrated no significant differences in mortality or long-term complications. Both methods significantly reduced HFH, but LBBAP resulted in a significantly shorter QRS duration, higher postoperative LVEF, and shorter procedure and fluoroscopy times compared to BVP. LBBAP proved to be a better method than BVP for CRT as a first-line therapy [92].

In the randomized controlled LEVEL-AT trial conducted on 70 patients over a 6-month observation period, the outcomes of CSP-CRT (conduction system pacing for cardiac resynchronization therapy) and BVP-CRT (biventricular pacing for cardiac resynchronization therapy) were observed. The primary endpoint was the change in left ventricular activation time, while secondary endpoints included left ventricular remodeling, HFH, and death due to heart failure. The results showed that CSP-CRT and BVP-CRT were similarly effective in changing left ventricular activation time, as well as in left ventricular remodeling and the incidence of complications. This makes CSP-CRT a viable alternative to BVP-CRT [93].

In recent years, a survey was conducted in Europe to obtain an up-to-date picture of current CSP practices. The survey involved 171 cardiologists. It was found that, for patients with LBBB and HF, traditional CRT remained the first-line treatment. For patients with atrioventricular block and in cases where the implantation of a lead in the coronary sinus failed, CSP was most often indicated. In most cases, LBBP was preferred over HBP due to high stimulation thresholds and the inability to reverse the bundle branch block. In cases of HBP, the use of a right ventricular lead was rare and mainly occurred in cases of pacing and ablation. The results of the survey indicate that most operators reserve CSP for cases of CRT failure in the group of patients with heart failure and LBBB [94].

### 10.1. Impact of Gender on the Effectiveness of CRT

The impact of gender on LBBAP outcomes in CRT has also been studied [95]. It was found that, compared to men, women experience significantly greater reductions in the composite endpoint of death or HFH with LBBAP-CRT compared to BVP-CRT.

One theory posits that the phenomenon of a better response to CRT among women is due to smaller heart sizes and greater relative dyssynchrony for a given QRS duration (QRSd). A post hoc analysis of the MORE-CRT MPP study provided evidence of a significantly higher response rate to CRT for women compared to men. The regression analysis of the group with non-ischemic cardiomyopathy and LBBB showed that it is not gender but QRSd/LVEDV that is the modifier of response to CRT, and this parameter was significantly higher among women. The relationship between response to CRT and QRSd/LVEDV was strongest for QRSd < 150 ms [96].

### 10.2. New Predictive Parameters of CRT Efficacy

Global longitudinal strain (GLS) is a better indicator for predicting adverse cardiac events than LVEF measurements because it is not dependent on external factors and has high repeatability, serves as an independent predictive factor for left ventricular reverse remodeling, and for patients in sinus rhythm, the optimal cut-off value is −10% [122]. Biventricular pacing (BVT-CRT) was compared with HBP-CRT in terms of its impact on mechanical dyssynchrony and longitudinal systolic function among patients with HF, LVEF ≤ 35%, and LBBB. The assessment was made before performing CRT and again 6 months later. Changes in mechanical dyssynchrony were evaluated by comparing the standard deviation of time-to-peak in 12 midventricular and basal segments, while changes in longitudinal systolic function were assessed using changes in GLS. Mechanical dyssynchrony significantly decreased in both groups after 6 months (*p* < 0.001); similarly, GLS showed significant improvement in both groups (*p* < 0.001), but no significant differences were found between the groups. Comparable effects of LBBP, BVP, and HBP-CRT were achieved in heart failure patients in terms of mechanical dyssynchrony and GLS [97].

## 11. Remodeling in Pacemaker-Induced Cardiomyopathy

One common complication of standard right ventricular pacing is pacemaker-induced cardiomyopathy (PICM). PICM can be prevented through BVP. HBP and LBBP mimic natural conduction and are characterized by even lower inter-ventricular dyssynchrony [98]. It appears that HPCSP improves cardiac function by synchronizing the electrical and functional activity of the heart, resulting in the reversal of ventricular remodeling. In one meta-analysis [99], it was shown that the stimulation of the pacing system in patients with PICM resulted in a shortening of the QRS complex from 175 ± 19 ms (*p* < 0.001) during right ventricular pacing to 116 ± 18 ms (*p* < 0.001) after upgrading to HPCSP; LVEF improved from 35 ± 8% to 48 ± 12% after upgrading to HPCSP (*p* < 0.001); and NYHA class decreased significantly from 2.7 ± 0.8 to 1.9 ± 0.8 (*p* < 0.001). CRT prevents the deterioration of LVEF and worsening of heart failure in patients with normal or mildly reduced LVEF who require high levels of ventricular stimulation [100].

Chen et al. [101] proposed a single-center randomized controlled study of 46 patients with PICM to compare the outcomes of His-Purkinje system pacing (HPSP) with traditional CRT. The evaluation will include QRS duration, levels of NT-proBNP, C-reactive protein (CRP), antibiotic therapy used, LVEF, LVESV, LVEDV, HFH, postoperative infection rate, 6 min walking test, pacing threshold, and mortality. The research team will assess the efficacy and safety of HPSP treatment in the group of patients with PICM and compare the efficacy and safety of this treatment with CRT. Evidence is needed that could lead to the clinical application of HPSP in patients with PICM.

Currently, there are no specific recommendations for modifying pacing methods in individuals who already have an RV pacing device and experience worsening LV function. Transitioning to BVP or CSP is recommended only when LVEF is less than 35%. A comparative study of RVP to modified pacing methods using a lead placed in the coronary sinus, His bundle, or left bundle branch showed improvement in LVEF, NYHA class, Minnesota heart failure score, and peak oxygen uptake with the modification of standard RV pacing. Similar effects were noted among CRT modifications. Complications for CRT modification within 24 months included 2% for pneumothorax, 1.4% for tamponade, and 3.7% for infection. These risks are outweighed by the benefits of CRT methods among patients with LV dysfunction [102].

## 12. Technical Aspects of Conduction System Pacing

The HBP electrode can be successfully placed in only 80% of cases, often due to the inability to fix the electrode in a location with a reasonable capture threshold. The site for placing the pacing electrode is determined using the traditional point-by-point trial-and-error method, often utilizing the pacing electrode. The difficulty in determining the optimal pacing site to stimulate the conduction system and overcome the bundle branch block makes it challenging to precisely locate the optimal site for stimulation. HBP and LBBP are technically complex methods performed by a narrow group of experienced electrophysiologists. These procedures are conducted under the guidance of two-dimensional fluoroscopy and electrograms. Orlov et al. [103] described a new technique for HBP using electroanatomic mapping (EAM). They conducted a study with 28 patients. EAM included mapping the atrioventricular septum, His bundle, and selective and non-selective HB capture sites. The EAM system was connected to the pacing leads, which were navigated to the designated HBP sites, and then the leads were placed in the locations indicated by EAM and marked on the His bundle area cloud. The group of patients achieved repeatable lead navigation to the HBP sites indicated by EAM. The His bundle cloud was divided into three segments, and no correlation was found between the electrode location in the His bundle cloud and the HBP threshold.

Bhatt et al. [104] aimed to evaluate the outcomes of HBP in real-world clinical conditions. Although RVP increases the risk of heart failure, HBP requires the reprogramming and upgrades of devices, which present significant practical challenges. At Valley Hospital in Ridgewood, New Jersey, the success rate of HBP implantation was 75%. A significantly lower effectiveness of HBP was noted in cases of bundle branch block or complete heart block. The high effectiveness of HBP was recorded in atrioventricular blocks without bundle branch block. Limitations of the pacing technique include a high rate of increasing thresholds and lead revisions.

One of the prospective multicenter studies conducted from 2017 to 2018 evaluated the efficacy and feasibility of LBBP-CRT using a new intraseptal technique in a group of patients with LBBB, nonischemic cardiomyopathy, and LVEF ≤ 50%. LBBP achieves electrical resynchronization by capturing the left part of the conduction system distal to the conduction block. The Select Secure lead was implanted from 1 to 1.5 cm along the axial line between the distal HBP site and the RV apex in the right interventricular septum. Subsequently, the electrode was placed deeply into the septum to achieve the capture of the left conduction system, gradually observing the appearance of terminal R-wave in lead V1 and an increase in impedance during single-pole stimulation. Low- and high-output stimulations were conducted to confirm left conduction system capture. The new two-electrode technique allowed for the assessment of Purkinje fiber activation during corrective HBP. The complete absence of pre-excitation Purkinje activation occurs with LBB. Corrective HBP is the only situation where Purkinje fibers can be mapped with LBB. Therefore, one electrode was placed at the His bundle location and the other inside the septum, providing direct evidence of Purkinje fiber activation during corrective HBP. In this study, the significant shortening of QRS duration, stable pacing thresholds, and R-wave amplitude; an increase in LVEF with a reduction in LVESD; the normalization of LVEF in 75% of patients after one year; and improvement in NYHA class were observed. No HFH or deaths were recorded during the follow-up. Stable and low pacing thresholds may offer an advantage over HBP for CRT [105].

Twelve-lead electrogram (ECG) is the gold standard for differentiating selective (S) and non-selective (NS) His bundle pacing and capturing the right ventricular septum, as it allows us to distinguish locations based on the morphology of HBP. A commonly reported problem with HBP is the loss of His capture, and the procedure is also very time-consuming. Currently, there are no algorithms to facilitate the HBP procedure. In a study [106], new practical criteria based on an electrogram (EGM) were proposed, which allow for the differentiation of selective and non-selective HBP and RV septal pacing. The criteria for determining the morphology of proper His bundle conduction are straightforward, but the assessment is challenging in patients with His bundle conduction disorders and BBB, as selective and non-selective His bundle capture can occur with or without bundle branch block recruitment. The following parameters were used to assess QRS complexes: near-field ventricular EGM morphology (NF EGM), near-field ventricular EGM time to peak (NFTime to peak), and far-field EGM QRS duration (FFQRSd). The morphology of the QRS complex was assessed in patients with indications for HBP, such as sinus node dysfunction, atrioventricular conduction disorders, and CRT. NF EGM was defined as the positive or negative initial sharp deflection of near-field EGM relative to the isoelectric baseline; NFTime to peak was defined as the time to the peak or nadir of positive or negative deflection of near-field EGM measured from the stimulus spike; and FFQRSd was measured from the beginning of the EGM (not the stimulus spike) to the peak or nadir of the terminal sharp deflection. NS-HBP causes the simultaneous activation of the RV septum and His bundle system, reflected in the immediate negative deflection of NF EGM after the stimulus spike, with a short time to the EGM peak. Conversely, in S-HBP, initial conduction after the stimulus spike occurs solely along the His bundle axis, which appears as the absence of an immediate negative deflection of NF EGM and a longer time to the EGM peak. The duration of FF QRS helps differentiate NS-HBP from S-HBP through the degree of QRS narrowing during conduction system pacing. Regardless of baseline QRS duration, high sensitivity (94% and 93%) and specificity (90% and 94%) for S-HBP were demonstrated by positive NF EGM and NFTime to peak > 40 ms, respectively. High negative predictive value (97%, 95%, and 92%) was shown by +NF EGM, NFTime to peak > 40 ms, and FFQRSd < 120 ms, respectively. EGM transitions correlated with ECG transitions, allowing for the precise differentiation of S-HBP, NS-HBP, and RV septal pacing. The cumulative predictive value was 91%. The new criteria may contribute to the safer and more efficient monitoring and observation of patients with HBP.

His bundle pacing addresses the problem of electromechanical dyssynchrony caused by right ventricular apical pacing. One study aimed to investigate whether direct HBP could be achieved through the conventional implantation of a pacing lead in various locations in the RV. In 2160 patients, the following locations were examined: RV apex, RV outflow tract, and mid-RV, with stimulation voltages of 2.5, 5, 7.5, and 10 volts (V). Regardless of the pacing location, an increase in stimulation voltage did not lead to changes in the morphology or duration of the QRS complex. There were no significant differences in QRS duration between the pacing sites or any advantage of one pacing site over the others. None of the examined pacing sites successfully captured the His bundle system [107].

A new imaging technique for HBP that involves the visualization of the tricuspid valve annulus was evaluated in a study on patients with indications for pacing [108]. In the study group, the tricuspid valve annulus was visualized by injecting contrast into the RV during fluoroscopy. The site for HBP was visualized near the tricuspid septal leaflet and interventricular septum. The control group consisted of patients who underwent HBP using the standard method. The study demonstrated significantly shorter fluoroscopy time and total procedure time with the new imaging method, which visualized the tricuspid septal leaflet, compared to the standard method. A quantitative relationship was also observed between the tricuspid septal leaflet and the HBP site. The method can identify the area of focus for His bundle pacing HBP on the atrial side (aHBP) and the ventricular side (vHBP). The aHBP area is associated with selective HBP, while the vHBP area is associated with non-selective HBP. The anatomical localization of HBP sites using contrast on the ventricular or atrial side of the tricuspid annulus is strongly associated with the physiological characteristics of pacing, which can help optimize lead placement according to the indications for pacing.

Many studies have evaluated the outcomes of HBP in expert centers with extensive experience. Given the lack of reports on the feasibility and safety of HBP performed by physicians without experience in conducting this type of pacing, a study was conducted involving the staff of three hospitals. Patients were selected for the procedure at the discretion of the physicians. HBP was successfully performed in 82.9% of patients, with selective HBP achieved in two-thirds of the patients and non-selective HBP in one-third. The study also demonstrated a rapid learning curve, with a success rate of 89.8% after 15 procedures. Thus, HBP proved to be a safe technique, which should encourage new operators to perform HBP in patients who are at risk for significant complications from RVP [109].

His bundle pacing poses challenges such as the low amplitude of the ventricular electrogram, high pacing thresholds, lead instability, and procedural complexity. In one publication, Cho et al. [110] described an alternative lead insertion trajectory. The new pacing technique was called cerclage parahisian septal pacing. It involved implanting a lead in the interventricular septum through the septal perforator branch of the great cardiac vein, which is the terminal branch of the coronary sinus. In their initial case description, the researchers achieved physiological pacing with cerclage parahisian septal pacing using a low pacing threshold with a bipolar heart sinus lead. They demonstrated the safety and feasibility of the new technique and showed a reduction in QRS duration with acceptable pacing thresholds, impedance, and lead sensing. The clinical protocol was terminated prematurely due to excessively large and poorly conducting commercial leads. However, the clinical experience led to the development of a newly designed pacing lead, the quadripolar cerclage pacing lead. This lead has three key features: (1) a low-profile monorail design with a tapered tip that allows for easier passage through the interventricular septum muscle; (2) narrow interelectrode spacing; and (3) a quadripolar design, which allows for the selectable stimulation of His or parahisian targets. This lead is easy to implant. The new methods avoid tricuspid valve regurgitation and provide physiological pacing and synchronous ventricular work. Further research is needed on new pacing techniques and alternatives to currently used pacing leads.

So far, LBBAP has been performed using lumen-less pacing leads (LLL) with a fixed helix design. In a prospective study, Pooter et al. [111] examined the feasibility and safety of LBBAP using stylet-driven leads (SDL) with an extendable helix design. The study demonstrated safety, a high success rate, a low complication rate, and low pacing thresholds for SDL.

Despite the many advantages of the new LBBP method, the implantation of the stimulating lead remains a challenge. In one study, Liu et al. [112] decided to investigate the feasibility of visualization-enhanced lead deployment for LBBP implantation. The study included 60 patients, half of whom underwent standard LBBP implantation, while the other half underwent LBBP using the image-guided technique. Using a visualization technique significantly reduced the procedure and fluoroscopy time and decreased the number of lead repositioning attempts.

The future for patients with ineffective conventional CRT might lie in wireless left ventricular pacing using the WiSE-CRT system [113]. This system consists of a transmitter placed above the intercostal muscle, connected to a generator implanted in the left mid-axillary line. A small electrode is implanted in the LV via a retrograde aortic or transseptal approach, and a co-implant capable of continuous RV pacing is also necessary. The co-implant sends a pacing signal detected by the transmitter, which then sends a focused beam of ultrasound energy to stimulate the endocardial electrode. The electrode converts the ultrasound energy into electrical energy, achieving simultaneous biventricular pacing. Implanting the wireless electrode on the LV septum instead of the lateral wall aids in LBBAP, extends battery life, and reduces the risk of ventricular perforation. To assess the safety and efficacy of this novel method, the WiSE-CRT system was tested on two pigs and subsequently on eight patients. The therapy proved effective, significantly shortening QRS duration, capturing the left bundle branch, and improving symptoms in 62.5% of patients. However, further studies are needed to evaluate the safety and efficacy of wireless septal LV pacing with the WiSE-CRT system.

Patients without LBBB, with ischemic cardiomyopathy, or with a QRS duration of less than 150 ms have a lower response rate to CRT compared to other patient groups. A novel surface mapping system, based on the standard deviation of left ventricular activation times, known as the ECG Belt System (EBS), was designed to measure electrical dyssynchrony. The question has arisen whether the use of EBS in lead placement and device programming will yield greater benefits than standard CRT care in this group of patients [114].

Optimal left bundle branch pacing requires an understanding of ventricular electrical heterogeneity (VEH). A modern technology used for this purpose is the ECG belt with multi-electrode ECG, and the measure of heterogeneity is the standard deviation of activation times (SDAT) from all electrodes in the ECG belt. SDAT is a predictor of response to CRT. Vijayaraman et al. [115] aimed to non-invasively assess VEH using the ECG belt to optimize LBBP in patients with bradycardia and heart failure. They assessed average left ventricular activation times (LVAT), SDAT of left-sided (LV dispersion) and right-sided (RV dispersion) electrodes, and changes in QRS duration (QRSd). All parameters were significantly reduced with LBBAP and LOT-CRT compared to native rhythm; however, no significant differences were found between selective, non-selective, anodal, and LV septal pacing. Regardless of the pacing mechanism, LBBAP reduced VEH, and the assessment of electrical heterogeneity using the ECG belt provided valuable information. The study is registered under the number NCT04583709.

Patients with a wide QRS complex and reduced left ventricular ejection fraction require a resynchronizing pacemaker or an ICD with a resynchronization function. In some patients with LBBB, who are in the group with the best electrotherapy outcomes, implantation of a His bundle pacing lead can be considered as a method to overcome LBBB. This approach leverages the known ability to bypass the site of conduction system damage and break the block. If intra-ventricular conduction disturbances cannot be resolved and the left ventricular/epicardial pacing effect is suboptimal, simultaneous pacing of the epicardial vein (a component of standard resynchronization) and the His bundle region can be used. This approach achieves better results in the form of a narrower QRS complex through the simultaneous activation of opposite walls of the left ventricle. It can be easily applied in patients with atrial fibrillation, where the port for the atrial lead is utilized for His bundle pacing. This method of resynchronization is referred to by the acronym HOT-CRT. The HOT-CRT method has become an alternative to BVP in CRT. There is evidence of better improvement in LVEF and a reduction in LVAT with HOT-CRT compared to BVP-CRT. HOT-CRT significantly improves ventricular electrical synchronization compared to BVP and multipoint pacing (MPP) [116,117].

Since HBP-CRT does not always result in optimal QRS narrowing, Vijayaraman et al. [12] sought to determine if CRT could be optimized through sequential HBP followed by left ventricular pacing (HOT-CRT) to maximize electrical resynchronization. In a study group of 27 patients with advanced heart failure and indications for CRT, HOT-CRT was effective in 25 individuals. The initial QRS duration significantly narrowed from 183 ± 27 ms with biventricular pacing to 162 ± 17 ms with biventricular pacing (*p* = 0.003), further to 151 ± 24 ms with HBP (*p* < 0.0001), and finally to 120 ± 16 ms with HOT-CRT (*p* < 0.0001). Significant improvements were observed in LVEF from 24 ± 7% to 38 ± 10% (*p* < 0.0001), NYHA functional class from 3.3 to 2.04, clinical response in 84% of patients, and echocardiographic response in 92% of subjects. HOT-CRT achieved enhanced electrical resynchronization of the heart.

The iSPOT study investigated the effectiveness of multiSPOT pacing (MSP) in improving the effects of BVP-CRT. MSP was performed using three electrodes on a four-chamber lead. Using hemodynamic parameters, A-V delay, QRS duration, and Q-LV, the efficacy of different pacing configurations was assessed: BiV with the LV lead placed in one of the lateral veins, with pacing from the distal, middle, or proximal lead, and MSP involving simultaneous pacing from three leads. LV multi-electrode pacing in patients with LBBB did not show an improvement in efficacy for conventional CRT, and it appears important to optimize atrioventricular delay for pacing efficiency [118].

In an another retrospective study, Vijayaraman et al. [119] aimed to evaluate the safety and efficacy of LBBAP using the Tendril 2088 lead introduced via a stylet-driven approach. The follow-up period was a minimum of 6 months post implantation. The primary endpoints included the absence of major adverse events related to the LBBAP lead, while the composite endpoint was defined as an LBBAP capture threshold ≤ 2.0 V and an R-wave amplitude ≥ 5 mV. The study reported that 99.5% of patients experienced no major adverse events related to the LBBAP lead, and 93% achieved the composite electrical performance success rate. The findings demonstrated the effectiveness and safety of the Tendril™ STS 2088 lead for LBBAP with a stylet-driven approach, showing a high success rate and a low complication rate.

The evaluation of the ST segment appears to be useful in assessing the correlation between myocardial injury current and the electrode’s action in HBP compared to LBBAP and RVSP. A positive myocardial injury current (IC) was defined as >0.2 mV or >25% increase in ST compared to the baseline value. The detection of positive changes in myocardial IC for the use of HBP was associated with a better threshold of capture, equal to that in LBBAP, both during implantation and in short-term follow-up [123].

## 13. Limitations

His bundle pacing requires meticulous electrode placement within the His bundle region, which can be technically challenging and demands advanced skills and specialized equipment. Anatomical variability or fibrosis can make the procedure even more difficult. Potential complications include perforation of the His bundle, damage to surrounding structures, and suboptimal capture or sensing due to improper electrode placement. The limited availability of specific electrodes and devices designed for HBP may restrict its use in some centers, and there may be a learning curve associated with their effective use. Not all patients with heart failure and conduction disorders are ideal candidates for HBP; for example, those with significant scarring or abnormal His bundle anatomy might experience less favorable outcomes. Long-term data on the effectiveness and safety of HBP across various patient populations remain limited. There is also a lack of long-term follow-up data regarding its durability and effectiveness, necessitating continuous monitoring and evaluation to assess long-term benefits and potential late complications. The technical demands and specialized equipment required for HBP can result in higher costs compared to traditional CRT, potentially affecting its accessibility and widespread use. Experience with HBP is still evolving, and the clinical evidence supporting it is less established compared to traditional pacing modalities. More comprehensive studies are needed to validate its long-term benefits and safety.

LBBP involves accessing the left bundle branch through a transseptal or retrograde approach, which can be technically demanding. Achieving optimal electrode placement for effective pacing may require intricate navigation and manipulation. Risks associated with LBBP include perforation or injury to the left ventricular myocardium or septum, as well as potential electrode dislodgement or malfunction. The procedure also carries a risk of inducing new conduction disturbances. Specialized electrodes and delivery systems for LBBP are not widely available and may not be accessible in all healthcare settings. Additionally, there is a need for the ongoing development and refinement of these technologies. While LBBP has demonstrated promising results, it may not be suitable for all patients, especially those with severe structural abnormalities or complex conduction disorders. Variability in pacing thresholds and response rates can affect outcomes. Similarly, long-term outcomes for LBBP are not yet fully established. Further studies are needed to determine the durability of clinical benefits and the risk of late complications. The cost of LBBP, including the need for specialized electrodes and devices, may be higher than conventional methods, which could impact its cost-effectiveness and availability in certain healthcare settings. Although LBBP shows potential, it is still a relatively new technique with evolving evidence. Additional research is required to define its role in standard clinical practice and refine patient selection criteria.

## 14. Conclusions and Future Directions

Physiological conduction system pacing methods, such as His bundle pacing, left bundle branch pacing, and the modifications of existing systems, offer potentially better clinical outcomes compared to traditional pacing systems. Through these innovative approaches, we can expect improvements in cardiac function and patient quality of life, while simultaneously reducing the long-term complications associated with pacing system implantation.

## Figures and Tables

**Table 1:** A total of 98 studies on the modern methods of cardiac electrostimulation were summarized.

Reference	Authors	Type of Study	Type of Pacing	Group	Short Summary
[24]	Muñoz et al. (PACE-FIB Study)	Randomized controlled trial	LBBAP + AVNA	Patients with HFpEF/HFmrEF and permanent AF	Clinical benefit associated with rhythm control efficacy using LBBAP with AVNA compared to pharmacotherapy.
[25]	Sefton et al.	Retrospective observational cohort study	LBBAP + AVNA	Patients with AF resistant to pharmacological therapy	Shows safety and efficacy of LBBAP with AVNA in advanced lung disease patients with AF.
[26]	Qian et al.	Meta-analysis	HBP	Patients with HF and cardiomyopathy undergoing AVNA	Demonstrates that HBP reduces QRS duration and improves LVEF in patients with cardiomyopathy and AF.
[27]	Zhang et al.	Prospective randomized controlled trial	His-Purkinje Conduction Pacing (HPCSP)	Patients with HFpEF and recurrent AF post-ablation	Evaluates HPCSP with AVNA for persistent AF and HFpEF, focusing on cardiovascular outcomes.
[28]	Huang et al.	Randomized controlled trial	HBP vs. BVP	Patients with persistent AF and LVEF < 40%	Compares HBP and BVP after AVNA, showing significant improvement in LVEF after HBP.
[29]	Wang et al.	Observational study	LBBAP	Patients with persistent AF and high rates of ventricular pacing	LBBAP impacts reverse remodeling of the left atrium in patients requiring high ventricular pacing.
			LBBAP vs. RVP	Patients with permanent AF requiring ventricular pacing and LVEF > 35%	Investigates LBBAP’s efficacy compared to RVP, showing significant improvements in LVEF and left atrial diameter.
[30]	Palmisano et al.	Multicenter observational study	LBBAP	Patients eligible for ablation and pacing	LBBAP shows shorter procedure time and lower thresholds compared to BVP and HBP with similar outcomes.
[31]	Palmisano et al.	Observational study	Ablation and CSP	Patients with persistent AF and drug-resistant high ventricular rate	Identifies predictors for spontaneous sinus rhythm restoration.
[32]	Chen et al.	Observational study	LBBAP	Patients with atrioventricular block (AVB) and HF	Shows lower risks of all-cause mortality, heart failure hospitalizations, and recurrent syncope with LBBAP compared to RVP.
[33]	Sharma et al.	Observational study	HBP	Patients with AVB, BBB and high rates of ventricular pacing	Demonstrates HBP’s utility as an alternative to BVP and a salvage strategy for unsuccessful BVP.
[34]	Wang et al.	Randomized controlled trial	HBP	Patients with AVB requiring pacemaker implantation	Evaluates LVEF, NT-proBNP, and quality of life metrics in AVB patients, with results currently being processed.
[35]	Cheng et al.	Randomized controlled trial	LBBAP	Patients with HFrEF and LBBB	Assessment of the stimulation threshold for LBBAP and higher R-wave amplitude along with easier implantation.
[36]	Ponnusamy et al.	Observational study	LBBAP	Patients with LV scarring	Evaluation of therapy success in patients with LV scarring using LBBAP.
[37]	Ponnusamy et al.	Observational study	LBBAP	Older patients with HF	LBBAP is safe and effective for patients over 80, improving echocardiographic parameters.
[38]	Mariani et al.	Meta-analysis	LBBP vs. BVP and HBP	Patients with HF and LBBB	LBBP significantly reduces HFH and has lower revision rates compared to BVP and HBP.
[39]	Abdin et al.	Meta-analysis	HBP and LBBP	Patients with bradycardia	HBP and LBBP maintain physiological ventricular activation better than RVP.
[40]	Leventopoulos et al.	Meta-analysis	LBBAP vs. RVP	Patients with bradyarrhythmia and conduction system disorders	LBBAP preserves synchrony and reduces HFH, AF occurrence, and mortality compared to RVP.
[41]	Vazquez et al.	Observational study	LBBAP vs. HBP	Patients undergoing CSP	LBBAP achieves better pacing parameters than HBP, with significant differences in capture thresholds.
[42]	Jastrzębski et al.	Observational study	LBBAP	Patients with bradyarrhythmia and HF	Demonstrates LBBAP efficacy in shortening QRS duration and reducing LVEDD, with varied capture types.
[43]	Qu et al.	Meta-analysis	HBP, LBBP, RVP	Patients with bradycardia and conduction disturbances	Shows RVP has fewer complications and shorter times, but leads to decreased LVEF and increased HFH compared to HPCSP.
[44]	Sun et al.	Meta-analysis	HBP vs. RVP	Patients with bradycardia and conduction disorders	HBP significantly improves LVEF and reduces pacing threshold compared to RVP, with lower HFH risk.
[21]	Slotwiner et al.	Meta-analysis	BVP and/or HBP vs. RVP	Patients with AVB and LVEF > 35%	Both BVP and HBP reduce LVESV and LVEDV while maintaining or improving LVEF compared to RVP.
[45]	Sharma et al.	Observational study	LBBAP vs. RVP	Patients with bradycardia	LBBAP shows better outcomes than RVP, including reduced QRS duration and lower HFH and mortality.
[46]	Zanon et al.	Observational study	HBP	Patients needing pacemaker implantation	Demonstrates the safety and efficacy of permanent HBP in various pacemaker indications.
[47]	Whinnett et al. (HOPE-HF trial)	Randomized controlled trial	HBP	Patients with HF, LVEF < 40%, prolonged PR interval > 200 ms, RBBB	No increase in LVEF; significant improvement in quality of life noted with optimized atrioventricular pacing.
[48]	Kronborg et al.	Randomized controlled trial	HBP	Patients with atrioventricular block (AVB), narrow QRS and HFpEF	HBP shows significantly better LVEF and mechanical synchronization compared to right ventricular pacing (RVSP).
[49]	Fernandes et al.	Meta-analysis	HBP vs. BVP vs. RVP	Patients with advanced AVB and HFpEF/HFmrEF	HBP and BVP significantly increase EF and reduce QRS duration compared to RVP, with lower mortality rates.
[50]	Sharma et al.	Prospective multicenter observational study	HBP	Patients requiring ventricular pacing	HBP showing higher thresholds but lower heart failure hospitalizations.
[51]	Vijayaraman et al.	Observational study	HBP vs. RVP	Patients needing pacemaker implantation	HBP shows lower incidence of pacing-induced cardiomyopathy and reduced HF hospitalizations.
[52]	Lustgarten et al.	Randomized controlled trial	HBP vs. BVP	Patients with HF	Equivalent response between HBP and BVP, with HBP demonstrating greater efficacy than LBBB.
[53]	Abdelrahman et al.	Observational study	HBP vs. RVP	Patients requiring permanent pacing	HBP significantly lowers the risk of death and hospitalizations compared to RVP.
[54]	Da Silva Menezes Junior et al.	Meta-analysis	HBP vs. BVP	Patients with HF	Shows HBP effectively shortens QRS complex and improves clinical outcomes.
[55]	Gin et al.	Meta-analysis	BB/LBBP	Patients undergoing CRT	Compares physiological pacing vs. BVP, showing improvements in QRS duration and LVEF.
[56]	Diaz et al.	Observational study	LBBP vs. LVSP vs. BIVP for CRT	Patients undergoing CRT	Highlights benefits of LBBAP over LV septal pacing with better outcomes.
[57]	Siranart et al.	Meta-analysis	LBBP	Patients with HFrEF and dyssynchrony	Confirms effectiveness of LBBP in reducing QRS duration and improving cardiac function.
[58]	Zhong et al.	Meta-analysis	LBBP for CRT	Patients who are candidates for CRT	Reports significant reductions in QRS duration and HFH with LBBP.
[59]	Parlavecchio et al.	Meta-analysis	LBBP-CRT vs. BVP-CRT	Patients with HF	Demonstrates lower HFH and improved clinical outcomes with LBBP-CRT.
[60]	Guo et al.	Observational study	LBBP-CRT	Patients with HF, LBBB, after CRT	Improvement in synchrony, LVEF, and NYHA function.
[61]	Shroff et al.	Multicenter study	LBBAP-CRT	Patients with HF, LBBB, after CRT	
[62]	Ferreira Felix et al.	Meta-analysis	CSP	Patients with HFrEF and dyssynchrony	
[63]	Salden et al.	Multicenter study	LV Septal Pacing	Patients undergoing CRT	LV stimulation can serve as a valuable alternative to CRT.
[64]	Vijayaraman et al.	Observational study	HBP and LBBAP	Patients undergoing CRT	Shows lower HFH and fewer deaths with CSP compared to BVP.
[65]	Kim et al.	Meta-analysis	CSP-CRT vs. BVP-CRT	Patients indicated to receive a CRT device	CSP reduces overall mortality and HFH compared to BVP-CRT.
[66]	Liang et al.	Comparative study	LBBAP vs. BVP for CRT	Patients eligible for CRT	Shows comparable impacts on morbidity and mortality for LBBAP compared to BVP in CRT.
[67]	Wang et al.	Meta-analysis	HPCSP vs. BVP for CRT	Patients indicated for CRT with HF	HPCSP improves clinical parameters and reduces HFH and mortality compared to BVP.
[68]	Cheng et al.	Meta-analysis	LBBP	Patients with HF and LBBB	LBBP significantly reduces QRS duration and improves LVEF and NYHA functional class.
[69]	Yu et al.	Meta-analysis	LBBAP	Patients with HFmrEF	LBBAP shows significant improvement in LVEF and QRS duration compared to traditional CRT.
[70]	Vijayaraman et al.	Multicenter study	LBBAP	Patients with HF and LBBB	LBBAP provides stable pacing thresholds.
[71]	Liang et al.	Crossover Study	LBBP vs. BVP for CRT	Patients with HF and LBBB	LBBP improves mechanical and electrical synchrony compared to BVP, showing significant hemodynamic benefits.
[23]	Wu et al.	Non-randomized observational study	LBBP, HBP, BVP	Patients with LVEF ≤ 40% and typical LBBB	Compares LBBP to HBP and BVP; both show better improvement in LVEF compared to BVP.
[72]	Abdin et al.	Meta-analysis	LBBAP vs. HBP	Patients requiring CRT	LBBAP superior to HBP in pacing parameters and implantation success rate as a first-line strategy.
[73]	Palmisano et al.	Prospective multicenter observational study	LBBAP vs. BVP for CRT	Patients with NYHA class III and IV and HFrEF	BVP associated with lower complication-free survival compared to LBBAP.
[74]	Su et al.	Observational study	LBBP	Patients with positive COI	In patients with positive COI, selective LBBP is more common, with stable stimulation parameters and no complications.
[75]	Jin et al.	Meta-analysis	LBBP vs. BVP	Patients with HFrEF and indications for CRT	LBBP shows shorter procedure and fluoroscopy times, greater QRS reduction, and improved LVEF compared to BVP.
[76]	Upadhyay et al.	Randomized multicenter trial	HBP	Standard indications for CRT	Compares His-CRT with BVP-CRT, showing greater QRS narrowing with His-CRT and no significant differences in mortality.
[77]	Chen et al.	Randomized controlled trial	LBBP	Patients with CRT pacemakers/ defibrillators	Evaluates LBBP effectiveness; shows significant LVEF improvement compared to BVP-CRT.
[78]	Ezzeddine et al.	Multicenter study	CSP	Patients with HF and indications for CRT	CSP resulted in greater improvement in LVEF compared to BiVP.
[79]	Peng et al.	Meta-analysis	LBBAP vs. HBP	Patients requiring a pacemaker	LBBAP may have advantages over HBP in stimulation thresholds, fluoroscopy time, and success rates.
[80]	Parlavecchio et al.	Meta-analysis	CSP	Patients requiring a pacemaker.	HBP has a lower complication rate compared to other pacing methods.
[81]	Tavolinejad et al.	Meta-analysis	CSP-CRT vs. BVP-CRT	Patients with HF	CSP-CRT shows greater improvements in QRS duration and echocardiographic parameters compared to BVP-CRT.
[82]	Wang et al.	Randomized controlled trial	LBBP-CRT vs. BVP-CRT	Patients with non-ischemic cardiomyopathy and LBBB	LBBP-CRT demonstrates greater improvement in LVEF compared to BVP-CRT.
[83]	Vijayaraman et al.	Observational study	LBBAP	Patients undergoing CRT	LBBAP significantly reduces QRS duration and improves LVEF compared to previous BVP treatment.
[84]	Al-Hennawi et al.	Meta-analysis	BVP, LBBP, HBP	HFrEF and indications for CRT	HBP shows shortest QRS duration, LBBP shows greatest improvement in LVEF and LVEDD.
[85]	Vinther et al.	Randomized controlled trial	HBP	Symptomatic HF and LBBB	HBP yields clinical improvement similar to BVP but with higher pacing thresholds.
[86]	Huang et al.	Observational study	HBP	HF with LBBB	HBP significantly increases LVEF, decreases LVESV, and improves NYHA class.
[87]	Sharma et al.	Observational study	HBP vs. BVP	RBBB patients with HF	Tests whether HBP is superior to BVP; HBP shows significant improvement in QRS duration and LVEF.
[88]	Chen et al.	Prospective multicenter observational study	LBBP-CRT vs. optimized BVP-CRT	HFrEF (EF ≤ 35%) and LBBB	LBBP-CRT shows shorter QRS duration and higher LVEF.
[89]	Gould et al.	Randomized controlled trial	Triventricular pacing	LBBB and intermediate QRS prolongation	Investigates triventricular pacing; shows no significant improvement compared to BVP-CRT.
[90]	Ajijola et al.	Multicenter study	HBP-CRT	Patients with HF	HBP-CRT leads to significant improvement in clinical and echocardiographic parameters, reducing QRS duration.
[91]	Strocchi et al.	Clinical trial	HBP, LBBP with optimized atrioventricular delay, BVP-epi, BVP-endo	CRT evaluation	HBP reduces LV activation times and improves dyssynchrony.
[92]	Diaz et al.	Observational study	LBBAP vs. BVP	Initial CRT strategy	Shows LBBAP is superior to BVP for CRT, with shorter QRS duration and higher LVEF.
[93]	Pujol-Lopez et al.	Randomized controlled trial	CSP-CRT vs. BVP-CRT	HF and wide QRS	CSP-CRT and BVP-CRT are similarly effective in improving LV activation time and remodeling.
[94]	Keene et al.	Randomized controlled trial	CSP	LBBB and HF patients	Shows CSP is preferred in cases where traditional CRT fails; LBBP is favored over HBP for pacing.
[95]	Subzposh et al.	Multicenter study	LBBAP-CRT	Patients receiving CRT	Women experience greater reductions in death or HFH with LBBAP-CRT vs. BVP-CRT.
[96]	Wijesuriya et al.	Randomized controlled trial	CRT	Non-ischemic cardiomyopathy and LBBB patients	Suggests response to CRT is influenced more by QRS duration and LVEDV than by gender.
[97]	Højgaard et al.	Randomized controlled trial	Biventricular Pacing (BVT-CRT) vs. HBP-CRT	HF patients with LVEF ≤ 35% and LBBB	Improvements in mechanical dyssynchrony and GLS without significant differences between groups.
[98]	Tokavanich et al.	Meta-analysis	HBP, LBBP	Patients with pacemaker-induced cardiomyopathy (PICM)	HBP and LBBP mimic natural conduction, reducing inter-ventricular dyssynchrony and preventing PICM.
[99]	Zheng et al.	Meta-analysis	HPCSP	Patients with PICM	Shows significant improvements in QRS duration, LVEF, and NYHA class after upgrading to HPCSP.
[100]	González-Matos et al.	Randomized controlled trial	CSP	Patients with HFmrEF and AV block	CRT prevents deterioration of LVEF and worsening HF in patients requiring high ventricular stimulation.
[101]	Chen et al.	Randomized controlled trial	HPSP	Patients with PICM	Compares HPSP with traditional CRT, assessing multiple cardiac parameters for efficacy and safety.
[102]	Kaza et al.	Meta-analysis	BVP, CSP	Patients with RV pacing and HFmrEF	Reports benefits of transitioning to BVP or CSP when LVEF < 35%, improving clinical outcomes despite risks.
[103]	Orlov et al.	Observational study	HBP	Patients requiring HBP electrode placement	Describes a new technique using electroanatomic mapping (EAM) for optimal HBP electrode placement.
[104]	Bhatt et al.	Observational study	HBP	Patients requiring right ventricular pacing	Evaluates HBP outcomes; success rate was 75%, with challenges in cases of bundle branch block.
[105]	Huang et al.	Multicenter study	LBBP-CRT	Patients with LBBB and nonischemic cardiomyopathy	Assesses LBBP-CRT, showing effective electrical resynchronization and improvements in LVEF and NYHA class.
[106]	Saini et al.	Multicenter study	HBP	Patients with indications for HBP	Introduces criteria for differentiating selective and non-selective HBP using electrogram (EGM) parameters.
[107]	Pang et al.	Clinical trial	HBP	Patients with indications for RVP	Investigates the feasibility of achieving direct HBP at various RV sites; none effectively captured the His bundle system.
[108]	Gu et al.	Randomized controlled trial	HBP	Patients with indications for HBP	New imaging method reduces fluoroscopy time and helps locate optimal HBP sites near the tricuspid valve.
[109]	Chaumont et al.	Multicenter study	HBP	Patients with indications for HBP	Shows HBP can be safely performed by less experienced operators, achieving a high success rate.
[110]	Cho et al.	Clinical trial	Cerclage Parahisian Septal Pacing	Patients with indications for HBP	Describes a new technique with low pacing thresholds and reduced QRS duration.
[111]	De Pooter et al.	Multicenter study	LBBAP	Patients requiring LBBAP with bradycardia or HF	Evaluates LBBAP using stylet-driven leads, demonstrating safety and a high success rate.
[112]	Liu et al.	Clinical trial	LBBP	Patients undergoing LBBAP	Investigates visualization-enhanced lead deployment, showing reduced procedure time and fewer repositioning attempts.
[113]	Elliott et al.	Multicenter study	Left Ventricular Septal Pacing	Patients with ineffective conventional CRT	Evaluates WiSE-CRT system, demonstrating effective pacing and symptom improvement in preliminary trials.
[114]	Rickard et al.	Randomized controlled trial	ECG Belt System	Patients with indications for CRT	Lack of effectiveness of EBS therapy in treating HFrEF with CRT.
[115]	Vijayaraman et al.	Clinical trial	LBBAP	Patients with bradycardia and HF	Assesses VEH using ECG belt to optimize LBBP; finds significant reductions in electrical heterogeneity.
[116]	Vijayaraman et al.	Randomized controlled trial	HOT-CRT	Patients with LVEF < 50% and indications for CRT	Evaluates HOT-CRT method, showing improved LVEF and reduced LVAT compared to BVP-CRT.
[117]	Zweerink et al.	Clinical trial	HOT-CRT	Patients with indications for CRT	Assesses sequential HBP followed by LV pacing, showing significant improvement in QRS duration and LVEF.
[118]	Sterliński et al.	Clinical trial	Multi-SPOT Pacing (MSP) for CRT	Patients with LBBB needing CRT	MPS-CRT provides a comparable improvement in contractility to BVP-CRT.
[119]	Vijayaraman et al.	Multicenter study	LBBAP	Patients receiving LBBAP with the Tendril 2088 lead	Evaluates safety and efficacy of LBBAP using the Tendril 2088 lead.

All abbreviations used in the table are explained in the main text.

## Data Availability

Not applicable.
